# Evaluation of two implementation strategies in 51 child county public service systems in two states: results of a cluster randomized head-to-head implementation trial

**DOI:** 10.1186/s13012-014-0134-8

**Published:** 2014-10-14

**Authors:** C Hendricks Brown, Patricia Chamberlain, Lisa Saldana, Courtenay Padgett, Wei Wang, Gracelyn Cruden

**Affiliations:** Department of Psychiatry and Behavioral Sciences, Northwestern University Feinberg School of Medicine, 750 North Lake Shore Dr, 10th Floor, Chicago, IL 60611 USA; Oregon Social Learning Center, 10 Shelton Mcmurphey Blvd, Eugene, OR 97401 USA; University of South Florida, 13201 Bruce B Downs MDC 56, Office 2130, Tampa, FL 33612 USA

**Keywords:** Implementation, Multidimensional Treatment Foster Care, Stages of Implementation Completion

## Abstract

**Background:**

Much is to be learned about what implementation strategies are the most beneficial to communities attempting to adopt evidence-based practices. This paper presents outcomes from a randomized implementation trial of Multidimensional Treatment Foster Care (MTFC) in child public service systems in California and Ohio, including child welfare, juvenile justice, and mental health.

**Methods:**

Fifty-one counties were assigned randomly to one of two different implementation strategies (Community Development Teams (CDT) or independent county implementation strategy (IND)) across four cohorts after being matched on county characteristics. We compared these two strategies on implementation process, quality, and milestone achievements using the Stages of Implementation Completion (SIC) (Implement Sci 6(1):1–8, 2011).

**Results:**

A composite score for each county, combining the final implementation stage attained, the number of families served, and quality of implementation, was used as the primary outcome. No significant difference between CDT and IND was found for the composite measure. Additional analyses showed that there was no evidence that CDT increased the proportion of counties that started-up programs (i.e., placed at least one family in MTFC). For counties that did implement MTFC, those in the CDT condition served over twice as many youth during the study period as did IND. Of the counties that successfully achieved program start-up, those in the CDT condition completed the implementation process more thoroughly, as measured by the SIC. We found no significant differences by implementation condition on the time it took for first placement, achieving competency, or number of stages completed.

**Conclusions:**

This trial did not lead to higher rates of implementation or faster implementation but did provide evidence for more robust implementation in the CDT condition compared to IND implementation once the first family received MTFC services. This trial was successful from a design perspective in that no counties dropped out, even though this study took place during an economic recession. We believe that this methodologic approach of measurement utilizing the SIC, which is comprised of the three dimensions of quality, quantity, and timing, is appropriate for a wide range of implementation and translational studies.

**Trial registration:**

Trial ID: NCT00880126 (ClinicalTrials.gov).

**Electronic supplementary material:**

The online version of this article (doi:10.1186/s13012-014-0134-8) contains supplementary material, which is available to authorized users.

## Background and objectives

One of the leading scientific challenges in implementation research is to determine what strategies are optimal to implement evidence-based programs successfully in communities, organizations, and populations. There is a considerable debate in the field about the best scientific approaches for answering such questions; one approach suggests that multiple baseline and other non-randomized designs often are the most appropriate or most acceptable [1,2], while others argue for the use of randomized implementation trials that contrast implementation strategies against each other [3–8]. This paper is based on the analysis of a randomized implementation trial of an evidence-based behavioral intervention delivered through county public child service systems. We first present new analytic measures and models for testing differences in speed, quality, and quantity of implementation between two different strategies, each of which targets implementation of the same evidence-based program, Multidimensional Treatment Foster Care (MTFC) [9], an alternative to group or residential placement.

Specifically, we tested whether a peer-to-peer Community Development Team implementation strategy for county child public service systems (CDT; experimental condition) could improve the speed and quality of implementation, the quantity of families who received MTFC, and the ability of counties to reach competence in continued delivery of MTFC [10] compared to the existing individualized, single independent county implementation strategy (IND) or comparison condition. As a type of learning collaborative, CDT required counties to work together to develop their respective implementation plans and overcome barriers in implementation, whereas counties in IND developed their own plans and worked individually with the MTFC purveyor (i.e., as is typically done in MTFC implementation efforts). These two implementation strategies are compared to one another, holding fixed the same evidence-based intervention.

MTFC, a behavioral mental health intervention program, was implemented in both conditions. MTFC involves placement in specially trained and supported community-based foster care homes in lieu of placement in aggregate care settings. MTFC is a top-tier evidence-based intervention for high-need youth in the child public service systems, including juvenile justice, mental health, and child welfare systems. This program is one of the original ten highest tier evidence programs designated by the Blueprints for Healthy Youth Development whose certification standards for determining which programs are evidence-based are among the highest in the field [11]. MTFC has demonstrated effects on preventing violence, delinquency and criminal behavior, illicit drug use, depression, and teen pregnancy, and because of these benefits and its high potential for cost savings [12], it has been identified by many states in the United States as one of a handful of evidence-based programs to be implemented as an alternative to restrictive placement in residential or group care settings. California, for example, certified MTFC as one of only two programs that they approved for addressing the needs of high service using foster care children [9].

This paper compares two alternative ways to implement MTFC. All counties in this trial were randomized to either CDT or IND and participated between 3 and 6 years based on assigned cohort in this rollout design [7]. In contrast to standard efficacy and effectiveness trials where the key interest is on evaluating improvement in health outcomes for the ultimate target population, this study focused on the changes that occur in the multilevel public service systems responsible for implementing MTFC. The levels range from leaders (county directors) in the mental health, child welfare, and juvenile justice systems to the private agency directors implementing the model, their clinical teams of front line practitioners, and the foster parent who is directly responsible for the care of the foster child.

The central question being addressed in the current trial is whether implementation success was enhanced by participation in the CDT. From a system perspective, implementation success involves process (e.g., how well counties prepare to deliver MTFC) and output (e.g., how many families served). In our specific situation, we ask whether those counties that were randomly assigned to the CDT condition deliver MTFC to a greater number of eligible youth with better quality and speed of implementation, and with more competence (e.g., reach full credentialing) compared to IND. Because implementation involves multiple attributes (e.g., quality, quantity, and speed) and multiple milestone attainment (e.g., first child placed in MTFC home, full credentialing), we use as our primary outcome a composite score based on three important components. The composite measure involved the number of implementation stages attained by a county, the number of families that received MTFC, and the sum of all quality indicators that were completed across all implementation stages. Our primary hypothesis was that CDT-assigned counties would score higher on this composite score than IND-assigned counties. In terms of secondary outcomes, we predicted that the number of counties that successfully delivered MTFC would be greater under CDT than IND conditions. We further hypothesized that those in the CDT condition would reach competency in implementing MTFC more often than those in IND. Third, we expected that a greater number of youth would be placed into MTFC under CDT than under IND conditions. Among these individual outcomes, the number of youth placements (i.e., penetration) into MTFC by the end of the study period was considered the most relevant to measuring successful implementation.

## Methods

### Overview of the trial design

Trial recruitment began in California in May 2006. The trial was expanded to include Ohio counties to the participating California counties from March 2009 through November 2010. Follow-up ran from approximately March 2007 to April 2012 in California, and from June 2009 to May 2010 in Ohio. All outcome measures for this study were derived from the Stages of Implementation Completion [13–16], which measures implementation processes, including how complete an implementation occurred, how fast it occurred, the quality with which it was implemented, number of clients served, and which milestones were achieved, including program start-up and credentialing. The trial involved a head-to-head comparison of the peer-to-peer CDT and individualized IND strategies.

### Trial participants

As we describe in more detail below, counties that had prior experience implementing MTFC were excluded from the trial; thus, this study focused on “non-early adopting” counties [17]. In the years prior to this study, the California Institute of Mental Health (CiMH), which provides technical assistance, research, and policy development to California counties to implement evidence-based practices, embraced the use of MTFC for the state and extended a general invitation to all California counties to receive training in MTFC. At that time, a total of 9 of California’s 58 counties elected to participate.

### Eligibility

This project used two exclusion criteria in selecting counties across the two states: those that had received MTFC previously and those that were too small to make MTFC a viable program. First, counties could not have been early adopters of MTFC. Thus, the nine California early adopting counties mentioned above were excluded from the trial. The County of Los Angeles also was excluded from randomization due to a class action lawsuit which led to a decision to require that MTFC/CDT be used in this county. Secondly, the remaining non-early adopting counties with too few foster youth eligible for MTFC were excluded from this study. Specifically, we excluded counties that had six or fewer youth in care on two snapshot days during the prior year, as this number was too small to maintain an active MTFC referral flow. This size restriction excluded an additional eight low-need counties. The remaining 40 eligible California counties were targeted for recruitment and randomization in 2006.

Two years into the project, recruitment was extended to Ohio to increase sample size. Due to study resource limitations, only an additional 12 counties were sought for recruitment. Using virtually identical inclusion/exclusion criteria as applied to the California counties, i.e., removing counties with prior MTFC implementation efforts and fewer than 6 youth in care, 38 of 88 Ohio counties were deemed eligible. These counties were randomly ordered and approached for participation in the trial, intending to recruit 12 counties from Ohio. However, only 11 counties agreed to take part during the time-limited open recruitment period we offered. Therefore, a total of 51 counties were recruited for participation.

### Recruitment

At the time of each state’s respective recruitment, all eligible counties were sent an invitation letter to participate in this study, explaining that the project would evaluate two different strategies for implementing MTFC: IND implementation and CDT. It was explained that IND-assigned counties would work singularly, as is customarily done with MTFC implementation (i.e., business as usual), while the CDT-assigned counties would engage in peer-to-peer networking and problem solving with five to seven other counties. The letter also explained that each county would be randomly assigned to one of these two implementation conditions in order to evaluate which implementation method was most effective in yielding successful implementation outputs.

### Outcomes: the Stages of Implementation Completion

The Stages of Implementation Completion (SIC) [10] measure was developed to evaluate completion, speed, and quality of implementation progress in both the CDT and IND conditions. The SIC defines eight stages represented within three phases of implementation (i.e., pre-implementation, implementation, and sustainability^a^) and includes the measurement of activities that involve interactions at multiple levels during the implementation process including system leaders, agency directors, practitioners, and clients (Table 1).

**Table 1:** **Stages of Implementation Completion (SIC) by agent involved**

**Phase**	**Stage**		**Involvement**
Pre-implementation	1	Engagement	System leader
	2	Consideration of feasibility	System leader, agency
	3	Readiness planning	System leader, agency
Implementation	4	Staff hired and trained	Agency, practitioners
	5	Adherence monitoring processes in place	Practitioners, youth/family
	6	Services and consultation begin	Practitioners, youth/family
	7	Ongoing services, consultation, fidelity monitoring and feedback	Practitioners, youth/family
Competency	8	Certification/competency	System, agency, practitioner

In the current study, the SIC includes steps that have been identified as essential to the successful adoption, implementation, and attainment of competency for the MTFC model. Similar to most evidence-based programs, MTFC follows a manualized protocol that includes numerous organizational and planning tasks, as well as well-specified intervention strategies [9].

### Components of SIC used in addressing hypotheses

The SIC measure was used to generate three types of scores used in forming the primary composite outcome; the final stage attained in the implementation process (0–8), the total of all activities indicating quality of implementation across stages 1–8—indicating how thoroughly or completely implementation was carried out and the number of families receiving MTFC. From these, we computed the composite score used in analyses for our primary hypothesis. We adjusted each of these three measures for cohort since their observation times were different. We then scaled the residuals to have a variance of one and used the first principal component - the linear combination of the data having maximal variance - to form the composite score used in analyses.

For secondary hypotheses, we examined each of the three separate measures described above as well as the number of stags attained and when two major milestones occurred, when a county first placed a youth, and when clinical competency occurred.

Previous evaluation of the SIC by Saldana and Chapman has demonstrated strong psychometric properties [16] and ability to predict meaningful implementation outcomes such as successful program start-up [15].

### Implementation strategies as intervention conditions

We first describe the IND, used initially to implement MTFC. IND counties received the usual technical assistance and implementation support as is typically provided to teams who are adopting a new MTFC program. This includes a set of three readiness calls with a highly experienced MTFC purveyor and a face-to-face stakeholder meeting where the county stakeholders meet with the MTFC purveyor to ask questions, work through implementation procedures, and develop a concrete plan for start-up.

For IND counties - as well as CDT (see below for differences) - the readiness calls were followed by a 5-day all staff training for administrators, supervisors, therapists, and skill building trainers, a 2-day foster parent training, training in using the MTFC fidelity monitoring system, program start-up (placement of youth in MTFC foster homes), and ongoing consultation and support in implementing the model through weekly viewing of video recordings of foster parent meetings and consultation calls to maintain fidelity to the model.

Counties randomized to the CDT condition also received all of the activities in the previous paragraph. In addition, they received technical assistance from two CDT consultants who were trained and experienced in implementing the MTFC model as they had previously assisted the initial nine counties in California excluded from this study. This support was offered over the course of the implementation process in six peer-to-peer meetings and in monthly conference calls with program administrators. CDT facilitators either were the developers of the CDT model or trained by the CDT developers. These facilitators began engaging county leaders in the CDT condition around the decision to adopt MTFC using similar procedures as in the IND condition but explained that their county would begin meeting with five to seven other counties at a time to help problem solve and share information about implementation issues [18]. This group format allowed for sharing of ideas across teams, discussion of key barriers experienced by counties in California or Ohio that were unique to the state landscapes, and resource sharing. The CDT principles and elements have previously been described in detail [14].

The CDT learning collaborative can be considered as a special type of quality improvement collaborative (QIC). These QICs grew out of adaptations of early attempts to apply industrial continuous quality improvement processes to the organizational systems used to deliver these mental health programs [19–22]. CDT utilizes some of the same components as QIC models of implementation, particularly providing structured opportunities for collaboration and problem solving across sites. CDT also has unique features, including its focus on the adoption of evidence-based mental health programs by local public service agencies through the direction and support of a statewide mental health service. Further, most QIC models are intended to monitor patient level outcomes but sometimes fall short of measuring meaningful system level outputs that assess the extent of evidenced based program implementation [22].

### Randomization

**Figure 1:**
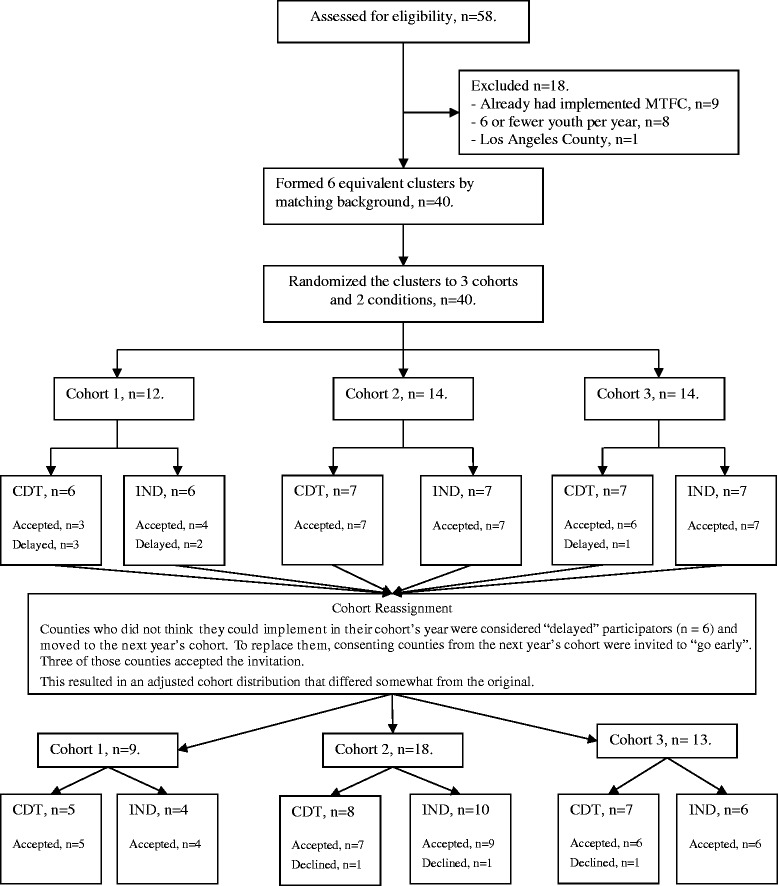
**Consort diagram for California counties.**

**Figure 2:**
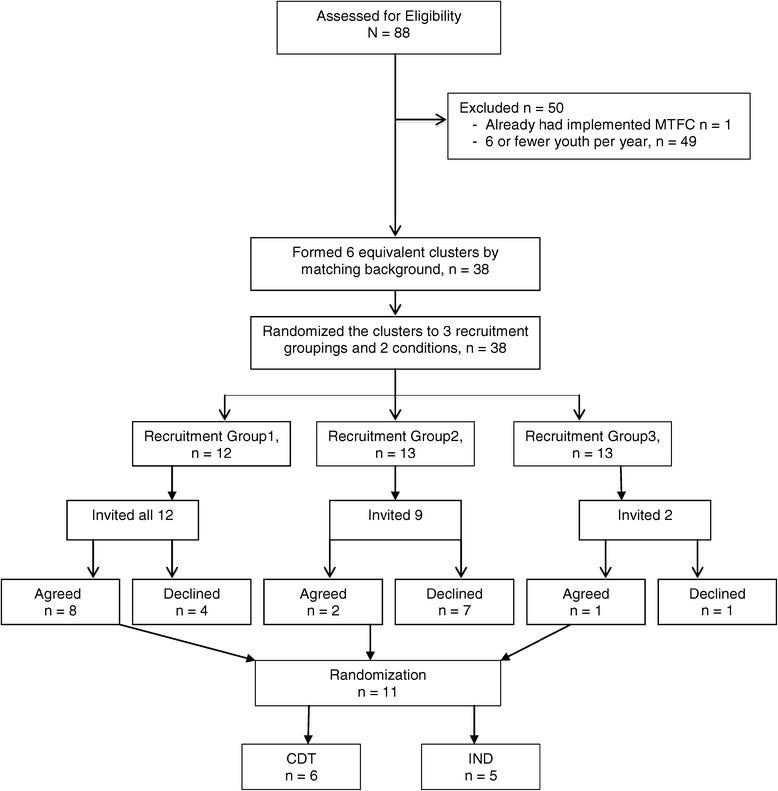
**Consort diagram for Ohio counties.**

In our protocol, we laid out a type of rollout design where randomization [4] of eligible counties would occur at two levels: implementation condition (CDT or IND) and when implementation would begin (three yearly cohorts, the last in two states). The second level of randomization involving timing was necessary to address study resource limitations and our inability to introduce the MTFC model to all counties at the same time. As per our protocol, eligible counties were matched within each state on county demographic variables including size, number of children in poverty, number of minority children, use of Medicaid, and per capita and group home placement rate. Although the matching criterion was the same across the two states, the randomization process differed slightly between the two (see Figures 1 and 2, consort diagrams). California counties were randomized prior to recruitment, as all eligible counties were invited to participate. On the other hand, because we recruited only 11 counties in Ohio, randomization occurred following their acceptance of the invitation to participate in order to maintain a balanced randomized design. A total of six equivalent groups of counties in California, each with six to eight counties, were constructed using random permutations of potential assignments of counties to cohort and condition so as to minimize between group differences on all county demographic variables among thousands of possible permutations of the same size. Thus, balance was achieved by assigning each of the 40 eligible counties in California to one of the 6 groupings of counties into cohort and condition, using the optimal assignment found through computer simulation.

### Allocation concealment mechanism

The six groups then were randomly assigned to cohort and to intervention through a computer-generated randomization programmed by the biostatistician. None of the counties, nor staff at the California Institute of Mental Health, were aware of their allocation until the research team informed all counties of their cohort and implementation strategy assignments. Randomization was determined similarly in Ohio, but there was only one cohort (year 3), so randomization only occurred for implementation strategy.

### Procedures to minimize contamination across implementation condition and allocation concealment method

A protocol for conducting this study was developed to minimize possibilities of implementation contamination across condition [7,9,17]. This was particularly relevant to prevent the IND assigned counties from receiving additional implementation guidance from CDT facilitators or other counties assigned to CDT, especially considering existing relationships that they had with these colleagues. Weekly meetings were held among the investigative team, IND and CDT facilitators, and the project biostatistician to track progress and limit contamination.

Because of the nature of the two implementation strategies, it was impossible to mask or blind these conditions, as well as cohort, from the counties or from the California Institute of Mental Health. The county leaders did know their county’s conditions, and they filled out online surveys themselves. Research staff collected concrete dates for each event in the SIC, and while they were not blind to implementation assignment, there was no decision-making in these tasks that could have introduced bias. Later in this paper, we describe how 9 of the 40 counties began at times not originally assigned. In replacing vacancies by moving counties to an earlier cohort while keeping their implementation condition constant, neither the California Institute of Mental Health intermediary/broker nor the counties themselves knew of the random ordering that the research team generated through a computer algorithm that we used to prioritize which counties were asked to move forward.

### Statistical methods

Our statistical analyses of this head-to-head randomized implementation trial were based on an intent-to-treat analysis in which the 51 counties’ responses were allocated to the assigned implementation strategy, whether or not the county completed any implementation stages.

We recognize that there are two types of clustering in this randomized trial, and it is known, but not sufficiently appreciated [23], that ignoring clustering can lead to incorrect statistical inference [24]. The first of these clusterings involves the four different cohorts of counties (three in California and one in Ohio) in the trial. Counties in the same cohort experienced the same times of follow-up and were exposed to the similar external forces. Because both implementation conditions occurred in each cohort (the statistical term is a blocking factor), this design was balanced across time and can therefore, with proper accounting of cohort as a random factor, yield appropriate inferences. Secondly, because of the peer-to-peer nature of CDT implementation, we would expect that the correlation among the CDT-assigned counties that work together to implement MTFC within a cohort could well be higher than that of the IND counties in the same cohort. This type of design is an example of an individual (here, county) randomized to receive treatment in a group design [24]. This second type of clustering often is ignored in such trials [23], but this oversight can also lead to inflated type I error rates, overly precise variance estimates, and spurious findings. Additional file 1 provides consort statements pertinent to this cluster randomized trial.

We used the random effect modeling [25] to handle both of these types of clustering. The most general model involved a separate random effect for the four CDT cohorts as in a cluster randomized trial and a separate random effect for the four CDT cohorts. If the random effect for CDT within cohort was found to be nil, i.e., the maximum likelihood estimate of intraclass correlation (ICC) =0, we considered a single cohort-level random effect that was comparable for both CDT and IND counties; that is, the 51 counties were nested into the four cohorts that shared a common variance, and counties were treated as having independent effects within these cohorts. If in addition, the random effect at the cohort level was found to be nil (cohort-level maximum likelihood ICC =0), we then reduced the model to a fixed effects model to account for potential variation across cohorts. Thus, in situations where the best point estimates of both cluster effects was zero, the 51 counties were considered as independent conditional on the cohort. If the point estimates were greater than zero, even if they were tiny, we reported this and included the random effects in our analysis. We also examined the effects of baseline covariates both as main effects and as having interactions with implementation condition. A final model was selected based on lowest Bayesian Information Criteria (BIC) [26].

We used censoring methods, common in survival analysis, to compare time to events. We included Cox proportional hazards modeling [27] to assess the total time each county took to make the first placement of a youth into an MTFC home, treating the time for those counties without any placement as right censored. A similar analysis was conducted on total time to reach stage 8, indicating the county had reached competency of program delivery. To compare the final stages attained between the two conditions, we used ordinal logistic regression [28] in order to estimate the proportional odds ratio of reaching higher stages. We chose ordinal regression over the Cox proportional hazard model since the latter is not robust when there are a large number of ties, as occurred with the scale containing only eight stages. We carried out mixed effects modeling using the lme4 and ordinal packages in R [25,29,30].

Prior to formal analysis, we examined the distribution of number of placements by graphical means. The data reflected a bimodal distribution with many counties having no placements (consistent with non-successful implementation) and the remainder had a wide range. We first compared the proportions of CDT and IND counties having any placements with Mantel-Haenszel tests that were stratified by cohort and then compared the distributions of placements conditional on those counties having at least one placement. As the counts were highly skewed, we first tested whether a linear model, a generalized linear model (i.e., with a Gamma distribution), or a model that transformed the number of counts (i.e., logarithmic or square root) fit the data better using BIC as a criterion (after adjustment of the likelihood for transforming the dependent variable) [31]. This best fitting class of models was then used to carry out a test to compare the numbers of placements for CDT versus IND.

To analyze quality of pre-implementation, implementation, and competency separately, we summed the individual binary quality indicators then divided by the number of measures across stages 1–3 for pre-implementation, 4–7 for implementation, and 8 for competency in continuing to deliver MTFC to families. As these proportions all had positive scores, we examined a series of generalized linear models to account for positive skewness in the data. We again controlled for cohort in this analysis and adjusted standard errors and statistical tests for clustering of CDT counties within cohort.

In submitting this grant proposal, we projected that the proportion of agencies implementing MTFC in CDT counties would increase from 15%, as expected for IND, to 60%, anticipating a large benefit from CDT. Even if the ICC for CDT counties were very high, i.e., as large as 0.5, this design was expected to have 85% power. For outcomes that are more continuous, such as our composite measure used as our primary endpoint, we predicted that the trial would have sufficient power to detect an effect size of 0.9 at power 0.80 and an effect size of 0.8 at power 0.70 for a two-sided 0.05 level test given ICCs less than 0.10.

## Results

### Participant flow

Consort diagrams are provided for California counties (Figure 1) and for Ohio (Figure 2). Of the 58 California counties, 18 were excluded because they met exclusion restrictions; all remaining 40 counties consented to participate and were randomized. Of the 88 Ohio counties, 50 were ineligible and the remaining 38 counties were divided into three comparable clusters. A total of 12 Ohio counties were invited, 11 agreed to participate, and all of these were randomized.

### Baseline data

Counties that did not think they could implement in their assigned cohort’s year were considered “delayed” participators (*n* =6) and moved to the next year’s cohort. To replace them, consenting counties from the next year’s cohort were invited to “go early”. Three of those counties accepted the invitation. Thus, a total of 9 of the 40 cohort assignments in California were modified to fill vacancies in this rollout design (22%; 6 of 13 assigned to the first cohort moved to the second, 2 of 13 originally assigned to the second cohort were moved to the first, and 1 of 14 originally assigned to the third cohort was moved to the second, see Figure 1). Because of these cohort reassignments, we checked again for comparability of counties on baseline measures across the cohorts and the two implementation strategy assignments. All 40 California counties and all 11 Ohio counties retained their assigned implementation condition (IND or CDT); they only switched cohorts. Table 2 shows these comparisons. Assignment was balanced for cohorts as well as implementation condition, as there were no significant differences across the county demographic variables.

**Table 2:** **Baseline comparisons by cohort and intervention condition**

**Baseline variable**	**Cohort**	**Intervention**
Population 2006 (log)	2.482 (3, 47), *p* =0.928	−0.19 (0.348), *p* =0.587
Proportion in poverty	2.506 (3, 47), *p* =0.93	0.679 (1.527), *p* =0.659
EPSD^a^ rate ‘94-’95	0.561 (2, 37), *p* =0.425	−0.004 (0.005), *p* =0.457
EPSD rate ‘02-’03	0.299 (2, 37), *p* =0.257	0.001 (0.007), *p* =0.901
Entries into CW (log)	2.242 (3, 47), *p* =0.904	0.012 (0.36), *p* =0.974
Minority population (log)	1.281 (3, 47), *p* =0.708	−0.023 (0.488), *p* =0.963
Permanent placement	0.684 (3, 47), *p* =0.434	0.038 (0.062), *p* =0.55

^a^Early Periodic Screening, Diagnosis, and Treatment (EPSDT) Program.

### Recruitment

System leaders from (1) Child Welfare, (2) Juvenile Justice, and (3) Mental Health from each county were invited to consent to participate using the same procedures across implementation condition. Consent was only necessary from one of the three systems for the county to be included in the participant pool.

As previously noted, the recruitment procedures between California and Ohio varied slightly (see consorts). In California, all counties were recruited at the start of the study, but those in cohorts 2 and 3 were told that their start dates would be staggered annually. Thus, counties in these later cohorts could agree to participate without having to implement until 12 to 24 months later.

### Numbers analyzed in each cluster

In order to maximize our annual study resources, we reassigned cohorts in California (see consort diagram 1) to fill vacancies while maintaining assigned implementation condition and randomization. To do this, we first randomized the order of counties within their respective cohorts and then used this order to invite them to “move up” in the rollout, always replacing counties who wanted to delay with those in the same implementation condition. This process allowed us to be responsive to county needs, while also maintaining our randomization and balanced design. Of the six CDT-assigned CA counties originally in the first cohort, three of these were reassigned and began with cohort 2. Of the seven IND CA counties originally assigned to the first cohort, three of these were reassigned and began with cohort 2. Two of the seven CDT counties originally assigned to cohort 2 were reassigned and began with cohort 1. One of the seven IND counties originally assigned to cohort 3 was reassigned and began with cohort 1. This resulted in 9 Californian counties in cohort 1 (5 CDT, 4 IND), 18 in cohort 2 (8 CDT, 10 IND), and 13 in cohort 3 (7 CDT, 6 IND). Because Ohio counties were added to the sample during California cohort 3, there was no need to delay or “move up” start dates in this state. Therefore, it was necessary for Ohio counties to consent to participate at the time of invitation or not at all. We randomized 11 Ohio counties into cohort 4 (6 CDT, 5 IND).

### Outcomes and estimation: primary hypothesis

For our composite measure that combined the total number of stages attained, the number of youth receiving MTFC services, and the quality of implementation across all eight stages, a principal component analysis on these standardized measures revealed a nearly equal weighting of all three measures for the first principal component. A linear regression analysis of this first principal component on implementation condition, using random effects for cohort and CDT groups found a positive but non-significant effect of CDT versus IND (effect size =0.24, CI = (−0.35,0.83), *p* =0.42).

### Outcomes and estimation: secondary hypotheses

For the secondary hypotheses, the three variables included in the composite score were analyzed separately, and we also tested whether the proportion of counties that successfully started up a MTFC program, the proportion of programs that reached clinical competency, and the speed of implementation (i.e., timing to first placement) differed by implementation.

### Comparing the total number of stages attained

**Figure 3:**
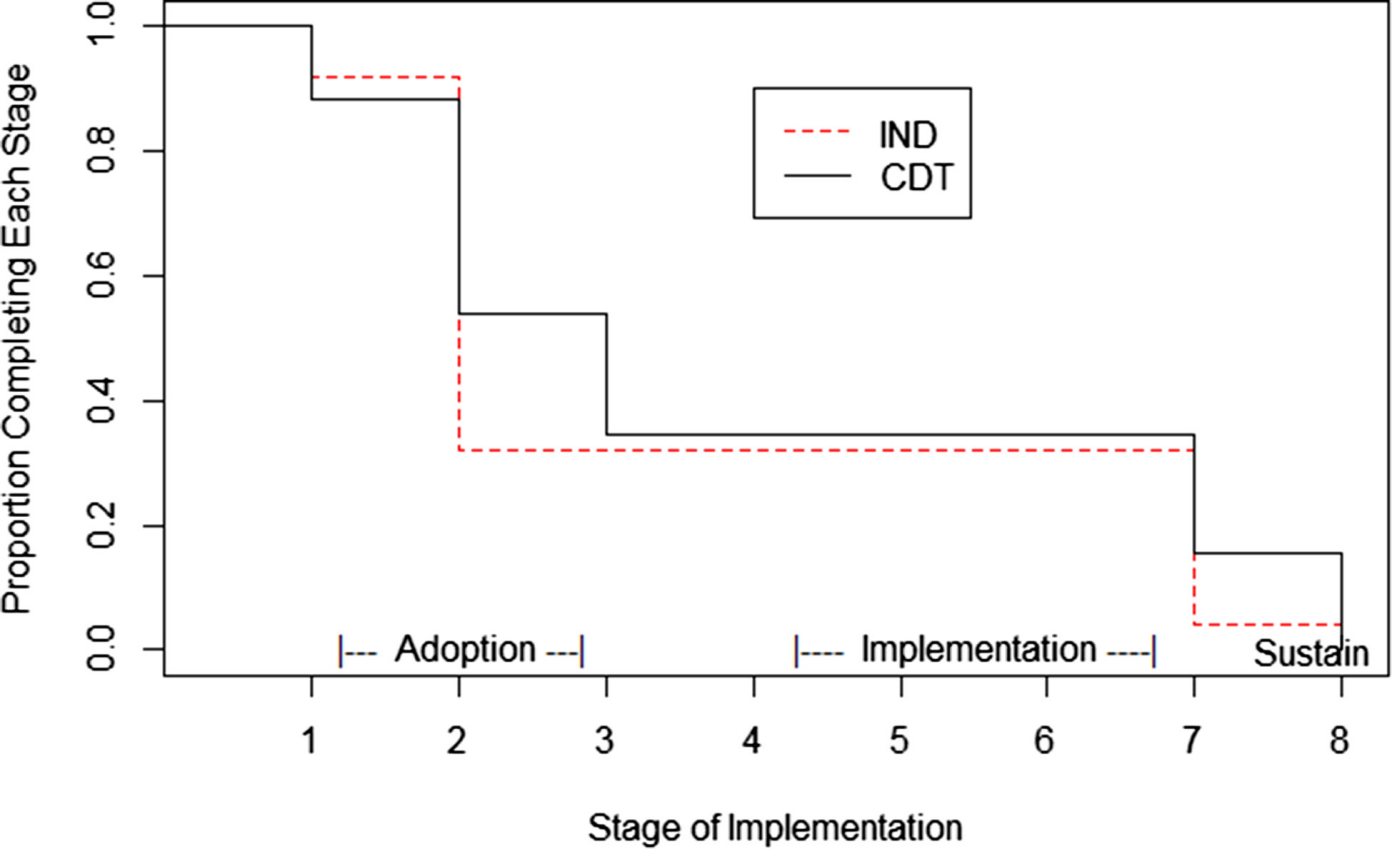
**Final stage of implementation for community development team (CDT) and independently administered (IND).**

For both CDT and IND, counties’ final implementation stage ranged across the full eight stages. Figure 3 shows Kaplan-Meier type curves corresponding to highest stage attained rather than the more traditional time to an event. Beyond stage 2, the small separations in the curves suggest that the CDT counties were slightly more likely than IND to reach higher stages based on an ordinal logistic regression analysis, but this did not reach significance when stratifying by cohort (proportional odds ratio =1.35, 95% CI = (0.46, 3.95), *p* =0.59). Both the median (three for CDT and two for IND) and the unadjusted mean (3.96 for CDT and 3.52 for IND) were slightly higher for CDT compared to IND. Using a cumulative ordinal mixed effects regression model, we found no difference in the final stage attained by implementation strategy (*p* =0.39). We also found that implementation strategy was non-significant in a fixed effect analysis that adjusted for county need (number of placements on snapshot days) cohort, and state (mean difference in CDT versus IND =0.23, 95% CI = (−0.90, 1.37), *p* =0.68). There was no indication of any interaction of cohort by implementation strategy (F(3,43) =1.12, *p* =0.35), although the California counties in the last two cohorts passed through significantly fewer stages than those of the first cohort and the only Ohio cohort.

### Comparing the number of youth receiving MTFC services

**Figure 4:**
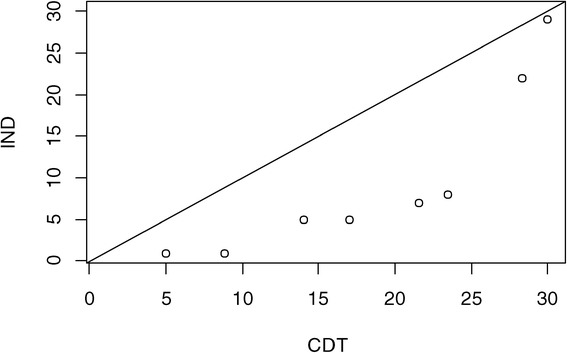
**Comparison of placement quantiles for CDT and IND counties.**

Overall, the average number of placements in MTFC per site over the study period was 6.4 for the CDT-assigned counties, compared to 3.1 for IND. This difference is not significant overall when tested in a quasi-likelihood Poisson model with fixed cohort effects (*p* =0.27). However, as previously noted, the proportions of counties that had no placements was nearly identical (*p* =0.94); thus, we examined the distribution of numbers of placements between CDT and IND only among those counties that placed any youth. The differences in distribution of placements are represented in an exploratory fashion through an empirical quantile-quantile plot in Figure 4. Each quantile for CDT is plotted against that for IND; the fact that all quantile comparisons fell at or below the diagonal line of equivalence indicates that the CDT distribution of placements appears to have higher values for each quantile (e.g., median, upper and lower quartiles) than that for IND. We followed up this exploratory examination by searching for a best fitting model to the data.

The distribution of the number of placements was far from normally distributed, so we compared the fit under a number of different transformations and families of distributions. Among all the models we tested, treating the square root transform as normally distributed had a BIC score that was 2.3 units lower than that of other models. We therefore chose to analyze the data on the square root scale. We examined whether county demographic characteristics or cohort contributed to the model; neither was significant, although the intraclass heterogeneity due to cohort was just slightly above zero (ICC =10^−9^). The effect of CDT was marginally significant (coefficient =1.42, CI = (−0.01, 2.86), *p* =0.051 on 12 *df*), indicating that there were higher numbers of placements under CDT versus IND. Among counties that did place a youth into MTFC, those randomized to CDT placed nearly 2.5 times the number of youth as those in IND.

### Comparing the proportion of counties that successfully started up MTFC

Nine out of 26 counties in the CDT condition placed one or more youth (35%), and 8 out of 25 counties in the IND condition placed one or more youth (32%). Controlling for cohort, there was no significant difference between conditions (Mantel-Haenszel odds ratio 0.95, CI = (0.24, 3.72), *p* =0.94).

### Comparing the proportion achieving competence

Achieving competency is the first step toward achieving sustainability, and this starts to occur at Stage 8 on the SIC. Five of the 51 counties received full certification during the study time period, indicating competency. Of these five, four were in the CDT condition, but there were no significant differences by implementation strategy (odds ratio =4.06, p =0.43 by Mantel-Haenszel test).

### Comparing the timing of first placement and timing to obtain clinical competency

A formal Cox proportional hazard model predicting time to first youth placement in MTFC (which occurs at the beginning of stage 6), adjusting for level of county need and adjusting for cohort and state strata also yielded faster but non-significant differences for CDT versus IND (hazard ratio =1.13, 95% CI = (0.34, 3.79), p =0.84).

From Cox regression modeling, the speed with which CDT counties obtained competency was not significantly different from the IND county (hazard ratio =2.99, 95% CI = (0.35, 25.60), *p* =0.32).

### Proportion of pre-implementation activities completed

There was no evidence of any difference in quality of pre-implementation by condition using a random effects model (coefficient =0.04, CI = (−0.08, 0.16), *p* =0.65). Unlike our previous analyses, there was strong evidence of cohort effects on pre-implementation quality, with an intraclass correlation of 0.45, with cohorts 2 and 3 in California having significantly fewer pre-implementation activities completed compared to cohort 1.

### Proportion of implementation activities completed

An analysis was conducted based on the sum of activities completed throughout stages 4–7 to determine how thoroughly the counties completed the recommended implementation activities during the implementation phase. These values ranged from 8 to 19 for those 17 counties that began stage 4, all of whom completed stage 6. We found substantial intraclass correlation due to cohort effects (ICC =0.38). In the random effect model, we found that CDT had significantly higher overall quality of implementation scores for stages 4–7 than did IND (coefficient =0.126, CI = (0.01, 0.25), *p* =0.03).

### Proportion of competency activities completed

No differences were found between conditions regarding the number of activities completed in the competency stage. All five counties that reached this stage had the maximum score of 2.

## Discussion

In this study, we found no evidence that the CDT implementation strategy achieved higher overall implementation compared to that for IND using either a composite score or assessments of how many stages were completed, how fast they were achieved, whether a county achieved placement of any youth, or whether a county achieved full competency. These findings were dominated by an overall low rate of MTFC placement; 35% in the CDT condition and 32% in IND. Among those counties that did place at least one youth in MTFC, there were indications in both quality of implementation as well as quantity that CDT performed better than IND. Thus it appears that CDT’s effect was negligible in achieving first placement as well as speed or extent of moving through implementation states. CDT did, however, appear to increase the number of placements and increase the quality of implementation once implementation began. For these counties, CDT impact appeared to result in more robust MTFC programs as indicated by having significantly more youth placed in care during the study period among counties that began placements, and by having completed more implementation activities. None of the other secondary hypotheses were confirmed in this study.

### Generalizability

Findings from this trial on MTFC are likely to generalize to other counties and states since we focused on the 80% of counties that were non-early adopting. The only counties that were excluded from this study were those that had previous exposure to MTFC or served too few youth to make MTFC an economically viable program in these counties. The trial took place during one of the worst financial crises faced by states, yet the design and program implementation were maintained throughout.

We note that the California Institute of Mental Health played a critical intermediary role in this project in both states [32]. Other states may not have access to such an organization and may not have the state level infrastructure to carry out CDT. However, since the California Institute of Mental Health was successful in working with counties from another state, this technology is likely to be exportable.

### Limitations

Even with 51 counties in two states, this study of non-early adopting counties still had limited statistical power to examine some outcomes. Some of the non-significant findings might be affected by sample size issues; analyses showed some non-significant gains in implementation from CDT compared to IND. In particular, more CDT sites (*n* =4) were certified during the study period than IND sites (*n* =1), but this difference was not significant, and statistical power was clearly influenced by the overall low rate of certification. It is well recognized that the statistical power for time to event and binary measures (e.g., milestone attainment) have lower statistical power than do most analyses of continuous quantity and quality measures. Even for the primary analysis based on a composite measure whose distribution was close to normally distributed, the effect size was 0.28 while the *p* value was not close to significant (*p* =0.42). Such an effect size is considered small but could be meaningful in large-scale implementation. Also, despite the comparatively high cost and length of this randomized trial, the low numbers of cohorts made it very difficult to assess variation across time, especially for counties assigned to CDT. We may need to find unique opportunities when evidence-based interventions are being rolled out to share the expense of large-scale implementation trials.

Another limitation in this study is that we did not explicitly examine variation in the patterns of implementation across counties, but rather considered variation more as “nuisance” parameters in our analysis. Further analyses are needed to understand whether there are distinct patterns of implementation that occur (e.g., a long pre-implementation period), and whether such patterns can be predicted and therefore provide opportunities for individualized feedback and intervention with counties that are not making adequate progress. Such analyses are currently under investigation as part of an ongoing R01 (PI: Saldana).

It is not clear from our study whether using a different learning collaborative other than CDT to support the implementation of another evidence-based program would yield more or less improvements in implementation.

The cost of using the CDT approach is a major potential caveat because this cost is added to the business as usual costs enacted in the IND condition. Cost has high relevance to policy and system leaders and is being examined in additional analyses [16,33].

### Interpretations

The Community Development Team implementation strategy, which uses trained consultants with knowledge of local policies and conditions to guide problem solving in teams of counties facing similar implementation challenges, may be particularly important in implementing complex mental health interventions within social service settings, such as MTFC, in non-early adopting counties. In extending Rogers’ finding that innovations that can be implemented relatively simply have high potential for rapid diffusion, we find that a more intensive implementation strategy, such as CDT, is partially helpful when implementing complex interventions to less innovation-seeking organizations, such as the social service agencies responsible for delivering mental health interventions to the targeted populations [34]. The notion that routine face-to-face and telephone conferences allowing for peer-to peer exchanges with consultants who are knowledgeable about state and county conditions, regulations, policies, and politics seems intuitively obvious as a means to boost implementation prospects, but this is the first trial to definitively show some effects from this process. In fact, to our knowledge, this is the largest county-level randomized trial to compare two implementation strategies against each other to examine implementation effectiveness.

There are two dimensions where this study differs from other important implementation efforts involving evidence-based programs. First, the design of this study with its head-to-head randomized trial and the assessment of implementation success/failure using the SIC allows us to make empirical comparisons of distinct implementation strategies. CDC’s Dissemination of Effective Behavioral Interventions (DEBI) program [35], for example, used a national training center to provide training in HIV prevention programs to all community-based organizations that are interested, so there is little opportunity to test implementation effectiveness. Similar to the two arms of this implementation trial, DEBI provides training on the delivery of the program itself. Unlike the two arms in this study, DEBI does not provide much technical assistance pertaining to capacity building at the community level, nor on supervision once training has ended. By not including an implementation measure such as the SIC, the DEBI program has limitations in learning how important these additional steps are.

A second unusual feature is that CDT involves a peer-to-peer process of addressing challenges in delivering an evidence-based intervention. Many other implementation strategies rely solely on technical support delivered to a sole system. For example, the Blueprints Replication Initiative provided extensive capacity building support to communities to implement those top-tier programs that Blueprints had identified and whose providers also had, at that time, sufficient capacity to deliver implementation training [36]. In the Blueprint implementation project, they concluded that their training program led to high fidelity program delivery. Unlike our study, it is not immediately clear how many communities this Blueprints Replication Initiative initially contacted to participate in this study, nor was there any indication that implementation would be successful with any communities other than the early adopters.

We note that a broad-based prevention support system, Getting to Outcomes (GTO), has used a non-randomized design to evaluate their implementation strategy [37] in six organizations that received GTO support contrasting these to four that did not [37–42]. They reported no significant effect on self-efficacy across the two conditions but strong increases within GTO communities as a function of GTO participation. Three of the six hypothesized process components, evaluation-decision making, process evaluation mechanics, and continuous quality improvement mechanics showed higher scores among those sites that received GTO compared to control.

Our own trial provides important findings regarding the use of a particular learning collaborative, CDT, in implementing a mental health intervention within social service settings. While we have not decomposed the effects of the different components of CDT, the improvements in quality and quantity of implementation that we have found suggest limited optimism for the use of certain aspects of quality improvement collaboratives. In Nadeem et al.’s review of quality improvement cooperative research, they identified five randomized controlled trials of such implementation strategies, three of which used active control comparisons such as we have done [22]. This study thus contributes to this small but important literature.

In addition to providing some evidence for the hypothesized outcomes for CDT, this study also succeeded from a design point of view. The study used a novel rollout randomized implementation trial design in two states to compare two implementation strategies focused on one evidence-based mental health intervention. In this head-to-head randomized implementation trial, counties were randomized to both the timing of implementation and implementation condition. We had no difficulty obtaining consent from counties to participate, and throughout the design, we were able to keep counties true to their assigned condition. No county dropped out of this design once they began, although several elected not to implement MTFC. This is not completely surprising given that all of the participating counties in both conditions had previously been given opportunities to implement MTFC and they had declined to do so; these counties are described as non-early adopting counties.

The assignment of counties to cohorts allowed county leadership to plan in advance for implementation, and our protocol, which allowed counties to move to later cohorts and fill vacancies while remaining in the same implementation condition, provided sufficient flexibility for counties to make timing adjustments. Our protocol of weekly research meetings involving the CDT and IND consultants who supported implementation in both conditions minimized the potential contamination across conditions. More detailed social network analysis in the California counties demonstrated that trust and influence relationships between county leaders in the two conditions were similar and not likely to affect the conclusions of this study [43]. Finally, we note that this trial took place during a major economic recession, which did reduce the willingness of counties to implement a new program model. However, because of the randomized trial design, we could still make valid causal inferences comparing the two implementation strategies. Had this study been conducted under any design other than a randomized trial, we would not have been able to disentangle the effects of the extreme economic changes from the implementation condition effects.

We also note that this study introduces more sophisticated modeling of implementation processes than often is done. The SIC allowed us to measure implementation across multiple stages and milestones and across multiple levels of participants from county government to foster parents in the MTFC team. The SIC provided information on the quality and quantity of implementation as well. By assessing timing, quality, and quantity of implementation, we were able to pinpoint much more accurately what changes in implementation process occurred, including progress and lack thereof. We believe that this methodologic approach of measurement with a SIC scale of the three dimensions of quality, quantity, and timing is appropriate for a wide range of implementation studies. In a recently funded study, Saldana (R01 MH097748) is adapting the SIC for other child and family evidence-based programs for service sectors including schools, juvenile justice, and substance abuse treatment [16]. The purpose is to evaluate the common or universal implementation activities that are utilized across evidence-based programs in their implementation strategies and to examine whether these universal items are equally important in achieving implementation success. Similarly, the study examines if the stages of the SIC are stable across evidence-based programs even when the activities defining SIC stages might differ. These adapted SIC tools will then be evaluated for adequate psychometric properties, including predictive ability, in order to further examine the value of implementation process and milestones in achieving successful program implementation.

The SIC scale, as well as the analytic models described here, is also relevant to the field of translational research, which has focused particularly on milestone attainment and less on quality and quantity [44]. The traditional view of implementation as one single stage of translational research, concerning the “bedside to community” translation that begins with “bench” research, can be enriched and viewed from a broader perspective. Indeed, the SIC measurement system and the analytic methods described here, which were developed around implementation, could also be used to monitor the entire translational process from bench to bedside to community.

## Conclusions

In implementing an evidence-based mental health program, Multidimensional Treatment Foster Care, in 51 counties in two states, we conducted a head-to-head randomized implementation trial where counties were successfully randomized to one of two implementation strategies: the CDT or the IND. Using the Stages of Implementation Completion to assess the speed of milestone attainment and the quality and quantity of implementation, we found that CDT did not increase overall implementation based on a composite measure of stage attainment, number of youth placed, and quality of implementation. There was no difference in the proportion or rate of implementing MTFC compared to IND nor the speed of milestone attainment. Compared to IND implementing counties, CDT implementing counties served more than twice as many youth during the study period. Additionally, the quality of implementation was improved in these CDT counties.

## Endnote

^a^Additional competency certification is continuing beyond the formal end of the trial.

## Additional file

Additional file 1: Table S1. CONSORT 2010 checklist of information to include when reporting a cluster randomized trial.

## Abbreviations

CDT: Community Development Team strategy
ICC: intraclass correlation
IND: individualized, single-county implementation strategy
MTFC: Multidimensional Treatment Foster Care
SIC: Stages of Implementation Completion
QIC: quality improvement collaborative
